# Adverse effects of hydroxyethyl starch (HES 130/0.4) on intestinal barrier integrity and metabolic function are abrogated by supplementation with Albumin

**DOI:** 10.1186/s12967-016-0810-3

**Published:** 2016-02-27

**Authors:** Yuk Lung Wong, Ingmar Lautenschläger, Karina Zitta, Christin Schildhauer, Kerstin Parczany, Christoph Röcken, Markus Steinfath, Norbert Weiler, Martin Albrecht

**Affiliations:** Department of Anesthesiology and Intensive Care Medicine, University Medical Center Schleswig–Holstein, Schwanenweg 21, 24105 Kiel, Germany; Department of Pathology, Christian-Albrechts-University, Kiel, Germany

**Keywords:** Hydroxyethyl starch, Albumin, Resuscitation, Fluid substitution, Intestine, Intestinal barrier integrity, Erk1/2, Akt, I-FABP

## Abstract

**Background:**

Volume resuscitation with hydroxyethyl starch (HES) is controversially discussed and we recently showed that HES perfusion impairs endothelial and epithelial intestinal barrier integrity. Here we investigated whether Albumin containing HES solutions are superior to HES alone in maintaining intestinal barrier function.

**Methods:**

An isolated perfused model of the mouse small intestine was used to investigate the effects of: (i) 3 % Albumin (Alb), (ii) 3 % HES or (iii) 1.5 % HES/1.5 % Albumin (HES/Alb). Intestinal morphology, cell damage, metabolic functions, fluid shifts and endothelial/epithelial barrier permeability were evaluated. Potentially involved signaling mechanisms (Erk1/2, Akt and Stat5 phosphorylation) were screened.

**Results:**

HES induced histomorphological damage (p < 0.01 vs. Alb), by trend elevated the amount of luminal intestinal fatty acid binding protein and reduced galactose uptake (p < 0.001 vs. Alb). Luminal and lymphatic flow rates were increased (p < 0.001 vs. Alb), while vascular flow was decreased (p < 0.001 vs. Alb) during HES perfusion. HES also increased the vascular to luminal FITC-dextran transfer (p < 0.001 vs. Alb), pointing towards a fluid shift from the vascular to the luminal and lymphatic compartments during HES perfusion. Addition of Alb (HES/Alb) reversed all adverse effects of HES (p < 0.05 vs. HES), restored barrier integrity (p < 0.05 vs. HES) and improved metabolic function of the intestine (p < 0.001 vs. HES; p < 0.05 vs. Alb). Mechanistically, HES/Alb perfusion resulted in an increased phosphorylation of Erk1/2 and Akt kinases (p < 0.001 vs. HES), while Stat5 remained unchanged.

**Conclusions:**

Albumin supplementation abrogates the adverse effects of HES in the intestine and underlying mechanism may function via phosphorylation of Erk1/2 and Akt. Albumin containing HES solutions are superior to HES alone and may improve the suitability of HES in the clinic.

**Electronic supplementary material:**

The online version of this article (doi:10.1186/s12967-016-0810-3) contains supplementary material, which is available to authorized users.

## Background

Hypovolemia due to hemorrhage, burns, trauma, and dehydration either alone or in combination requires fluid therapy to restore vascular volume and normalize venous return, cardiac output and blood pressure. Therefore, the common clinical treatment for patients with hypovolemia is a fluid resuscitation using crystalloid or colloidal solutions [1, 2].

Beside human Albumin (Alb), hydroxyethyl starch (HES) a synthetic nonionic starch derivate available in different molecular weight and substitution forms, is one of the most effective and commonly used colloids in Canada, China, New Zealand, Switzerland and other non-European countries [1, 3]. However, based on several recently published studies that suggest a higher risk for renal replacement therapy and increased mortality after fluid resuscitation with HES [1, 4], the European Medicines Agency’s (EMA) Pharmacovigilance Risk Assessment Committee instructed that HES solutions must no longer be used to treat patients with sepsis, burn injuries or critically ill patients (EMA/606303/2013). Despite the EMA recommendation, HES is still used for the treatment of hypovolemia in various clinical settings throughout Europe and other countries.

Information about the HES mediated physiological and cellular mechanisms that are responsible for the adverse effects of HES in specific organs is very scarce and has mainly been described in the heart [5] and kidney [6]. Unexpectedly, animal studies even showed positive effects of HES on inflammatory processes during sepsis [7–10] and concerning the negative effects of HES solutions on vascular permeability in the heart, some studies suggested that addition of Alb to HES containing solutions might be beneficial and could have the potential to reduce the adverse effects of HES [5, 11]. A deeper understanding of the HES mediated mechanisms is therefore necessary and could help to reduce the adverse effects and augment the positive influence of HES in the affected organs under different clinical situations.

Endothelial and epithelial damage/dysfunction is a prerequisite for hypovolemia in most clinical settings. Therefore, in hypovolemia, fluid homeostasis is highly dependent on the function of the intestine, which offers a large inner surface area serving as a selective barrier between the organism and the environment [12]. The intestinal endo- and epithelia crucially preserve the fluid homeostasis and barrier function between the vascular, interstitial and luminal compartments. Various pathophysiological processes like inflammation or microbial sepsis result in an increased endothelial and epithelial permeability leading to edema formation as well as passage of pathogens and bacterial toxins into the systemic circulation [7, 8, 13–16]. Although the intestine plays a central role in metabolism and barrier function as well as in inflammation and sepsis, there are only few studies that have evaluated the effects of different fluid substitution strategies, specially with HES containing solutions, on intestinal function and barrier integrity [8, 9, 17, 18]. Employing an isolated perfused model of the mouse small intestine we have recently evaluated the intestinal effects of HES and showed that HES harms the intestine by increasing the endothelial and epithelial barrier permeability by so far unknown mechanisms [19].

Based on the above mentioned findings that Alb supplementation may have the potential to reduce the negative effects of HES in the heart and our own data that show adverse effects of HES in the intestine, we hypothesize that addition of Alb to HES containing solutions will attenuate the negative effects of HES on intestinal cell damage, metabolic functions, fluid shifts and endothelial/epithelial barrier permeability. Utilizing our perfused model of the small intestine we therefore evaluated histomorphological changes, apoptosis, I-FABP (intestinal-fatty acid binding protein) release, galactose uptake, lactate-to-pyruvate ratio, wet-to-dry ratio, fluid shifts and luminal FITC-dextran transfer after perfusion with 3 % Alb, 3 % HES or 1.5 % HES plus 1.5 % Alb. In addition phosphorylation of pro-survival kinases was investigated to obtain insight into the HES and Alb induced signaling events.

## Methods

### Isolated perfused mouse small intestine model

All experiments were performed with isolated small intestines from 7–9 weeks old C57BL/6 mice (Central Animal Facility UKSH, Kiel, Germany) housed with standard diet and water ad libitum before surgery [19]. The detailed surgical procedure and ex vivo perfusion has been described by our group recently [19]. Briefly, mice were anesthetized by inhalation of 1–3 % Sevoflurane and additional intraperitoneal injection of Ketamine up to a maximum dose of 40 mg/kg. The small intestine was dissected, isolated and perfused via the aorta and the intestinal lumen. Perfusion was performed with constant flow and changes in the vascular, lymphatic and luminal effluent weights, perfusion pressures and buffer compositions (O_2_, CO_2_, pH, electrolytes, glucose and lactate) were monitored as described previously [19].

### Experimental protocol

**Fig. 1:**
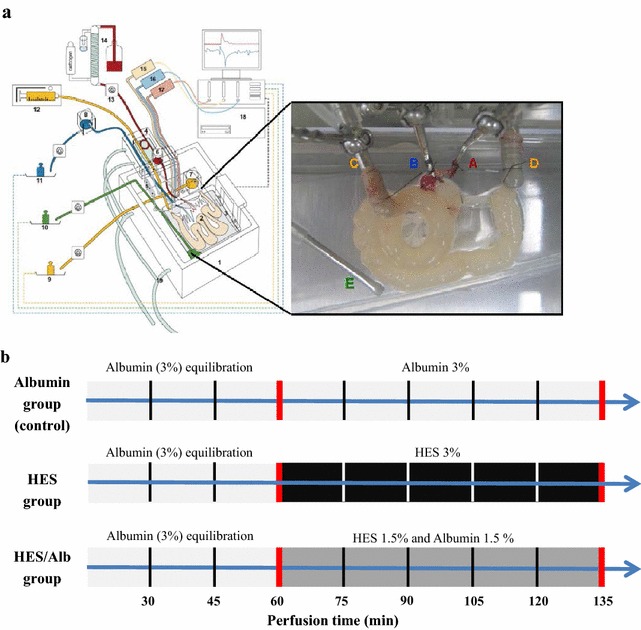
Experimental setting and basic components of the perfusion model. Isolated perfused mouse small intestine apparatus (**a**). Using a custom made, heated chamber (*1*) an isolated small intestine (*2*) is perfused (vascular system: *red*; luminal system: *yellow*) while placed on a built-in microbalance (*3*). A moveable cannulating block (*4*) carries the tubings, heat exchanger cannula holders (*5*), and bubble trap (*6*). Height-adjustable reservoirs (*7*, *8*) allow clamping both afterloads to zero. For online analysis of fluid homeostasis all emanating liquids are quantified by use of three balances (*9*–*11*). Constant flow perfusion is applied by a syringe pump (*12*) and a roller pump (*13*). The vascular perfusate is prewarmed, oxygenated, and pH equilibrated with a tempered hollow fiber dialyzer flushed with carbogen gas (*14*). Pressure transducers (*15*–*17*) allow online detection of the luminal (*yellow*), venous (*blue*) and arterial (*red*) pressures. All data are recorded on a personal computer (*18*). To secure constant temperature, the chamber and cannulating block are water-jacketed and warmed by a water bath (*19*). The *inset* shows a representative photograph of a perfused small intestine. *A* arterial cannula; *B* venous cannula; *C* oral intestinal lumen cannula; *D* aboral intestinal lumen cannula; *E* lymphatic suction needle; modified from [21]. Experimental setting and time frame (**b**)

Our recently described isolated perfused mouse small intestine model [19] was used to evaluate the physiological and cellular effects of a vascular perfusion with HES and whether the addition of Alb to the HES containing modified Krebs–Henseleit perfusion buffer is able to reduce the adverse effects of HES. The control group (Alb perfusion group, Alb, N = 6) received an initial vascular perfusion with Alb (3 %, Sigma-Aldrich, Hamburg, Germany) for 60 min (equilibration phase) followed by an additional 75 min of vascular perfusion with the same Alb containing buffer. The second group (HES perfusion group, HES, N = 6) received an initial vascular Alb (3 %) perfusion for 60 min (equilibration phase) followed by a perfusion with HES (3 %, 130/0.4, Fresenius Kabi, Bad Homburg, Germany) for 75 min. The third group (HES/Alb perfusion group, HES/Alb, N = 6) received an initial vascular Alb (3 %) perfusion for 60 min (equilibration phase) followed by a 75 min perfusion with HES (1.5 %) plus Alb (1.5 %; Fig. 1). All perfusions were continuously applied without intermittent stops. As colloid osmotic pressure substantially influences fluid distribution within the investigated compartments (vascular, interstitial, luminal) only the mentioned three preparations with similar osmotic pressures were employed. This was also the reason why perfusions with 1.5 % HES alone or 1.5 % Alb alone were not included into the study design.

### Evaluation of morphology, fluid shifts and metabolic function

Details about the respective methods have been published recently [19]. As an indicator for edema formation in the intestine, a calculation of the wet-to-dry ratio of the respective tissue was performed. Briefly, for the determination of the intestinal wet weight, a 3 cm long proximal portion of the small intestine without mesentery and intestinal content was obtained before and after perfusion. The dry weight was determined after dehumidifying the samples for 96 h at 55 °C. Additionally, at the end of perfusion a 3 cm long segment of the intestine was fixed in 4 % formaldehyde for histological examination in which sections of intestinal tissue were stained with hematoxylin–eosin and periodic acid–Schiff [20]. Analyses of tissue damage were performed by a blinded investigator employing a histological stability score which is based on the evaluation of epithelial integrity [21] with the exception that only longitudinal slices were evaluated. To determine the endothelial and epithelial permeability of the intestine, 40 mg/l FITC-dextran (150 kDa; Sigma-Aldrich, Hamburg, Germany) were added to the vascular perfusate. Samples of venous, lymphatic, and luminal outflow were collected every 15 min and analysed for the FITC-dextran content using a fluorescence ELISA reader (excitation 485 nm, emission 530 nm, Sunrise, Tecan, Crailsheim, Germany). Determination of galactose resorption was performed by supplying the luminal perfusion buffer with 30 mM lactose. Vascular galactose (derived from the luminal lactose) was determined by a commercially available assay kit (Raffinose/D-Galactose Assay Kit, Megazyme, Bray, Ireland). Vascular pyruvate was determined in the venous outflow by an enzymatic photometric method (NADH method, Sigma-Aldrich, Hamburg, Germany). For the measurement of vascular lactate a blood gas analyser was employed (Gem Premier 3000, Instrumentation Laboratory, Kirchheim, Germany).

### Determination of Caspase-3/7 activity

Activities of the effector Caspases-3 and 7, which play a central role in apoptotic events were evaluated in intestinal tissue samples before and after perfusion using rhodamine based fluorometric assays (Apo-One homogeneous Caspase-3/7 assay, Promega Corporation, Madison, WI, USA). Treatment of the samples and evaluation of Caspase-3/7 activity were done on the basis of the manufacturer’s protocol using a fluorescence ELISA reader (Tecan, Crailsheim, Germany) in combination with the Magellan software v1.1. Protein extraction was performed by using RIPA buffer containing 150 mM sodium chloride, 1 % NP-40, 1 % sodium deoxycholate, 0.1 % sodium dodecyl sulfate (SDS) and 50 mM Tris–HCl (pH 7.6; all from Sigma-Aldrich, Hamburg, Germany). The protein concentrations were determined with Roti^®^-Quant assays (Carl Roth, Karlsruhe, Germany).

### Western blotting

All Western blotting experiments were performed with intestinal tissue samples derived before and after perfusion with the exception of the I-FABP Western blot, for which luminal effluents were used. Protein extraction from intestinal tissue was performed with RIPA buffer containing 150 mM sodium chloride, 1 % NP-40, 0.1 % SDS, 1 % sodium deoxycholate, 50 mM Tris–HCl (pH 7.6; all from Sigma-Aldrich, Hamburg, Germany) with protease and phosphatase inhibitors (Roche, Mannheim, Germany), and the protein concentrations were determined with Roti^®^-Quant assays (Carl Roth, Karlsruhe, Germany). Samples were boiled for 5 min after addition of SDS polyacrylamide gel electrophoresis (PAGE) sample buffer (62.5 mM Tris–HCl, 2 % SDS, 10 % glycerol, 5 % β-mercaptoethanol, all from Sigma-Aldrich, Hamburg, Germany). Equal amounts of protein (30 μg) or luminal effluents (30 µl) were separated by 10 % SDS-PAGE and transferred onto a PVDF membrane (Amersham Pharmacia Biotech, Piscataway, USA). Membranes were then incubated in blocking solution (3 % BSA in TBS containing 0.05 % Tween 20; Sigma-Aldrich, Hamburg, Germany) for 1 h at room temperature, followed by an overnight incubation with specific antibodies against I-FABP (Abcam, Cambridge, UK; 1:500,000), Claudin-3 (Life Technologies, Darmstadt, Germany 1:1000), Erk1/2 (New England Biolabs, Frankfurt, Germany; 1:8000), phospho-Erk1/2 (New England Biolabs; Frankfurt, Germany; 1:8000), Akt (New England Biolabs; Frankfurt, Germany; 1:2000), phospho-Akt (New England Biolabs; Frankfurt, Germany; 1:1000), Stat5 (R&D Systems, Minneapolis, MN, USA; 1:1000) and phospho-Stat5 (R&D Systems, Minneapolis, MN, USA; 1:1000) or β-actin (Santa Cruz, Heidelberg, Germany; 1:2000) which served as a loading control. After washing in TBS containing 0.05 % Tween 20 (Sigma-Aldrich, Hamburg, Germany), the membranes were incubated for 1 h with horseradish peroxidase conjugated pig anti-rabbit immunoglobulin G (Dako, Glostrup, Denmark; 1:10,000), rabbit anti-goat IgG-HRP (Santa Cruz, Heidelberg, Germany; 1:10,000) or goat anti-rabbit-biotin (Abcam, Cambridge, UK; 1:10,000) followed by streptavidin-HRP (Serotec, Puchheim, Germany; 1:5000). The final reaction was visualized using enhanced chemiluminescence (ECL-Detection Reagents, GE Healthcare, Munich, Germany), and the membrane was exposed to x-ray film. Images were taken and the intensities of the respective bands were densitometrically analysed with the software ImageJ (v1.48v, NIH).

### Animal welfare and ethical statement

This study was carried out in accordance with the recommendations of the Guide for the Care and Use of Laboratory Animals of the National Institutes of Health. The experiments were approved by the local authority (Ministry of Agriculture, Environment and Rural Areas of the State of Schleswig–Holstein, Kiel, Germany; V311-7224.121-39). All studies involving animals are reported in accordance with the ARRIVE guidelines for reporting experiments involving animals.

### Statistical analyses

Based on our recently published results [19] a power analysis using GraphPad StatMate 2 for Windows (GraphPad Software, San Diego, USA) was performed to estimate the numbers of animals necessary to obtain significant results for the primary outcome parameters of fluid shifts and FITC-dextran translocation. All further statistical analyses were performed using GraphPad Prism 5 for Windows (GraphPad Software, San Diego, USA). All data sets were analysed for Gaussian distribution using the Kolmogorov–Smirnov test and -if applicable- evaluated using parametric statistics. Data are presented as mean values with standard deviations (SD). Statistical comparisons were performed using Student’s t-tests and one-way ANOVA with Bonferroni post-tests (for intra group comparisons) as well as two-way ANOVA with Bonferroni post-tests (for inter group comparisons). Differences were considered to be statistically significant if p was less than 0.05. Non-parametric data were analysed by Mann–Whitney-Tests, Kruskal–Wallis tests and Dunns post-tests.

## Results

### Histomorphology and cellular damage

**Fig. 2:**
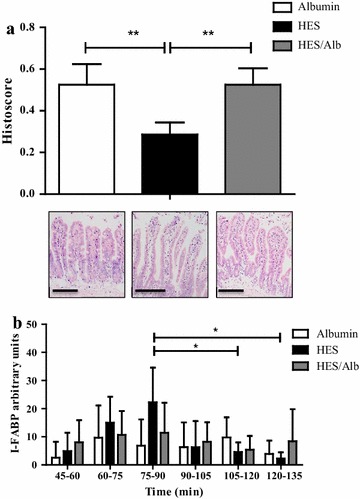
Histomorphology and release of I-FABP. Quantification of histomorphological damage and representative histological sections of the perfused intestine (**a**). Relative amounts of I-FABP in luminal effluents were estimated in 15 min intervals by Western blot analyses (**b**). *Bars* denote the mean ± SD. Albumin (N = 6), HES (N = 6), HES/Alb (N = 6). * p < 0.05; ** p < 0.01; *scale bar* 50 µm

A histological stability score [21] was modified as described in “Methods” section and used to evaluate the histomorphological damage of the intestinal epithelium. Perfusion with HES resulted in a significantly reduced histoscore compared to the Alb (p < 0.01; Fig. 2a) or HES/Alb group (p < 0.01; Fig. 2a). The histomorphological damage in the HES perfusion group was reflected by epithelial shedding and appearance of widened interstitial spaces within the intestinal villi. These changes were already detectable directly at the end of perfusion and could not be observed in the Alb and HES/Alb group (Fig. 2a). As a marker for damage of the intestinal epithelium, the amount of I-FABP was evaluated in the luminal effluents. During the first 90 min, an increased amount of I-FABP was detected only in the HES perfusion group. However, values did not reach statistical significance (p > 0.05; Fig. 2b). For the quantification of apoptosis, a Caspase-3/7 activity assay was employed. The results showed increased apoptosis in intestinal tissue that was obtained at the end of perfusion, but no differences in apoptosis between the groups. These findings are not surprising as the ex vivo model lacks a functional blood circulation and nerval innervation, which are both important for the survival of the respective tissue and cells (Additional file 1: Figure 1A). In addition, in vitro studies employing a commonly accepted model of intestinal epithelial cells (CaCo-2) revealed that even prolonged incubation with high concentrations of HES (3 %) did not induce apoptosis or cellular damage (Additional file 1: Figure 1B, C), suggesting that the adverse effects of HES on the intestinal epithelium in the perfusion model are rather related to an increased interstitial pressure (edema formation) caused by HES molecules within the interstitial compartment of the intestine and not due to HES-induced cytotoxicity or apoptosis.

### Basic metabolic parameters of the perfused intestine

**Fig. 3:**
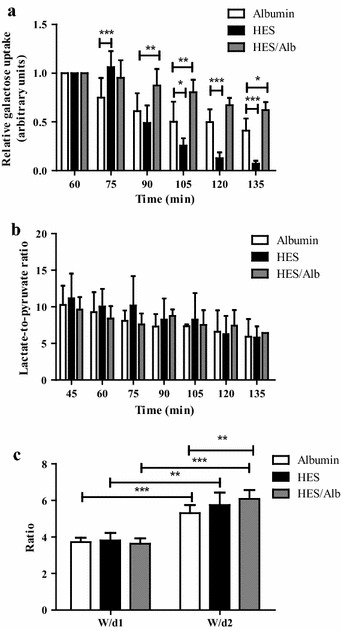
Metabolic parameters of the perfused intestine. The relative luminal galactose uptake was measured by a galactose assay kit (**a**), the lactate-to-pyruvate ratio was evaluated by blood gas analysis and an enzymatic photometric method (**b**), and the wet-to-dry ratio before (W/d1) as well as after (W/d2) perfusion was calculated (**c**). *Bars* denote the mean ± SD. Albumin (N = 6), HES (N = 6), HES/Alb (N = 6) for galactose and wet-to-dry ratio; Albumin (N = 2-6), HES (N = 6), HES/Alb (N = 1-6) for lactate-to-pyruvate ratio. * p < 0.05; ** p < 0.01; *** p < 0.001

Metabolic function of the intestine was evaluated by quantification of the galactose uptake (derived from luminal lactose) and the lactate-to-pyruvate ratio. Both parameters showed a time dependent decrease in all groups, which is related to effects of the ex vivo perfusion procedure on intestinal metabolism (Fig. 3a, b). However, intergroup comparison revealed a significant higher reduction of galactose uptake in the HES perfusion group at later time points compared to the Alb perfusion group (p < 0.05 for t105; p < 0.001 for t120 and t135; Fig. 3a). Interestingly, in the HES/Alb group the luminal galactose uptake was significantly increased compared to the Alb perfusion, suggesting positive effects of HES/Alb on metabolic functions of the intestine (p < 0.05 for t135; p < 0.01 for t90 and t105; Fig. 3a).

Regarding the lactate-to-pyruvate ratio, a perfusion related decrease was evident in all groups. There were no significant differences in the time response signatures within or between the groups (p > 0.05, intragroup and intergroup comparison; Fig. 3b) and all values were within the physiological range of aerobic metabolism [22]. In addition to the described metabolic parameters, the wet-to-dry ratio (W/d) was calculated. In all groups, W/d was higher at the end of perfusion (p < 0.001, Fig. 3c) and compared to a perfusion with Alb alone, W/d in the HES/Alb group was significantly increased (p < 0.01, Fig. 3c).

### Effects of different colloid containing solutions on vascular, lymphatic and luminal flow rates

**Fig. 4:**
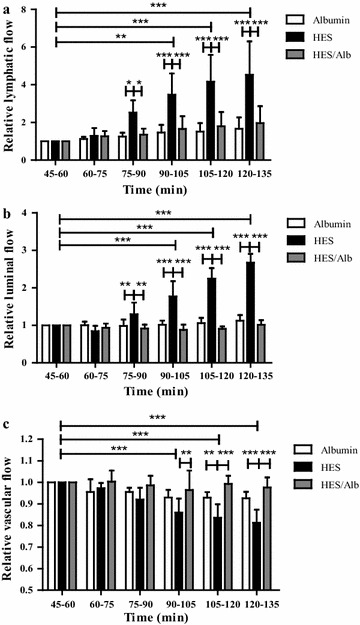
Effects of different colloid containing solutions on vascular, lymphatic and luminal flow rates. Lymphatic (**a**) luminal (**b**) and vascular flow rates (**c**) were measured by quantifying the effluent volumes every 15 min (t45–60 = 1). *Bars* denote the mean ± SD. Albumin (N = 6), HES (N = 6), HES/Alb (N = 6). * p < 0.05; ** p < 0.01; *** p < 0.001

For the vascular perfusion with HES the intragroup comparison showed a significantly increased lymphatic flow rate (p < 0.01 for t90–105; p < 0.001 for t105–120 and t120–135; Fig. 4a) and luminal flow rate (p < 0.001 for t90–105, t105–120 and t120–135; Fig. 4b), while the vascular flow rate decreased (p < 0.001 for t90–105, t105–120 and t120–135; Fig. 4c). Vascular, lymphatic and luminal flow rates did not show any statistically significant changes over time in the Alb and HES/Alb group (intragroup comparison, p > 0.05; Fig. 4a–c). Intergroup comparison (vs. the Alb group) revealed that HES perfusion significantly increased lymphatic flow (p < 0.05 for t75–90; p < 0.001 for t90–105, t105–120 and t120–135; Fig. 4a) and luminal flow (p < 0.01 for t75–90; p < 0.001 for t90–105, t105–120 and t120–135; Fig. 4b), while vascular flow was reduced (p < 0.01 for t105–120; p < 0.001 for t120–135; Fig. 4c) suggesting that HES perfusion impairs endothelial and epithelial barrier integrity, resulting in fluid shifts towards the lymphatic and luminal compartment. Replacing 1.5 % HES by 1.5 % Alb abrogated all negative effects of HES on lymphatic flow (p < 0.05 for t75–90; p < 0.001 for t90–105, t105–120 and t120–135; Fig. 4a), luminal flow (p < 0.01 for t75–90; p < 0.001 for t90–105, t105–120 and t120–135; Fig. 4b) and vascular flow (p < 0.01 for t90–105; p < 0.001 for t105–120 and t120–135; Fig. 4c). No statistically significant differences were detected between the Alb and HES/Alb group concerning lymphatic, luminal and vascular flow (p > 0.05; Fig. 4a–c).

### Effects of different colloid containing solutions on endothelial and epithelial barrier integrity

**Fig. 5:**
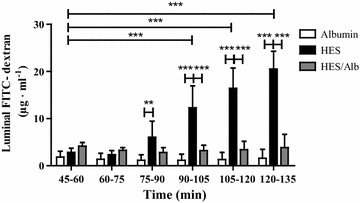
Effects of different colloid containing solutions on endothelial and epithelial barrier integrity. Luminal FITC-dextran concentrations were measured by fluorometry every 15 min. *Bars* denote the mean ± SD. Albumin (N = 6), HES (N = 6), HES/Alb (N = 6). ** p < 0.01; *** p < 0.001

As surrogate parameter for endothelial and epithelial barrier dysfunction, we determined the translocation of vascularly applied FITC-dextran into the luminal compartment. Perfusion with HES significantly and time dependently increased the luminal FITC-dextran concentrations (p < 0.001 for t90–105, t105–120 and t120–135; Fig. 5), whereas the FITC-dextran translocation into the luminal compartment remained unchanged in the Alb and HES/Alb group (p > 0.05; Fig. 5). Compared to Alb perfusion significantly increased luminal FITC-dextran concentration was observed during HES perfusion (p < 0.01 for t75–90; p < 0.001 for t90–105, t105–120 and t120–135; Fig. 5). Replacing 1.5 % HES by 1.5 % Alb abrogated the HES associated translocation of vascularly applied FITC-dextran into the luminal compartment (p < 0.001 for t90–105, t105–120 and t120–135; Fig. 5). Concerning the FITC-dextran translocation into the luminal compartment, no statistically significant differences were detected between the Alb and HES/Alb group (p > 0.05; Fig. 5).

Claudin-3 is an integral membrane protein and a component of tight junctions, which are forming continuous seals around cells, serving as a physical barrier to prevent solutes and water from passing freely through the paracellular space [23, 24]. Evaluation of the expression levels of Claudin-3 protein in the intestinal tissue derived after perfusion did not reveal any differences between the groups (p > 0.05; Additional file 2: Figure 2).

### Effects of different colloid containing solutions on phosphorylation of key signaling molecules

**Fig. 6:**
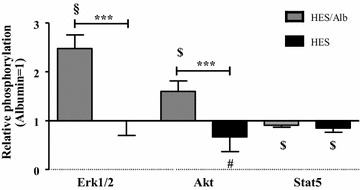
Effects of different colloid containing solutions on phosphorylation of key signaling molecules. Western blot analyses of Erk1/2, Akt and Stat5 phosphorylation in intestinal tissue lysates after perfusion. *Bars* denote the mean ± SD. Albumin (N = 6), HES (N = 6), HES/Alb (N = 6). *** p < 0.001; § p < 0.001 vs. Alb; $ p < 0.01 vs. Alb; # p < 0.05 vs. Alb

Compared to the perfusion with Alb, the phosphorylation of Erk1/2 and Akt was significantly increased in the HES/Alb group while Stat5 phosphorylation was reduced (p < 0.001 for pErk1/2, p < 0.01 for pAkt and pStat5; Fig. 6). In the HES group, only the phosphorylation patterns of Akt and Stat5 were reduced (p < 0.05 for pAkt, p < 0.01 for pStat5; Fig. 6). Comparing the phosphorylation of Erk1/2, Akt and Stat5 between the HES perfusion and the HES/Alb perfusion groups showed a significant increase of Erk1/2 and Akt but not of Stat5 phosphorylation in the HES/Alb group (p < 0.001 for pErk1/2 and pAkt, p > 0.05 for pStat5; Fig. 6).

Additional information about the measurements and raw data are also available in the Additional file 3.

## Discussion and conclusions

The application of HES for volume resuscitation is controversially discussed and clinical studies have suggested adverse effects of HES substitution, leading to increased patient mortality [1, 4, 25]. The intestine is of high clinical relevance and plays a crucial role in fluid homeostasis, sepsis and inflammation. Although some studies investigated the compatibility of colloids like Alb and HES in the perfused Langendorff heart [5] and kidney [6], information about the effects of HES on intestinal function and barrier integrity is very scarce and intestinal effects of HES containing solutions have so far not been investigated in vivo or using ex vivo systems. Therefore, our work is the first study that describes in detail the influence of HES, HES/Alb and Alb on intestinal metabolism, cell damage, fluid distribution and barrier integrity employing an isolated perfused model of the mouse intestine.

We have recently shown that vascular perfusion with a clinically relevant concentration of 3 % HES [26, 27] impairs the endothelial and epithelial barrier integrity of the small intestine [19]. Interestingly, the results of animal studies suggested that addition of Alb to HES containing solutions might be beneficial and may bear the potential to reduce adverse effects of HES [5, 11]. In the work presented here, we have therefore (i) reproduced and extended our previous ex vivo studies and investigated in more detail the physiological and cellular effects of a vascular perfusion with HES in the intestine, (ii) evaluated whether the addition of Alb to HES containing solutions is able to reduce the adverse effects of HES and (iii) screened for potential underlying mechanisms.

### Effects of different colloids on histomorphology, cellular damage and metabolic function

Analyses of tissue damage were performed with intestinal tissue that was obtained directly at the end of perfusion. A histological stability score which is based on the evaluation of epithelial integrity [21] was employed and revealed significantly increased histomorphological damage only in the HES perfusion group. Enterocyte coverage of the intestinal villi tips was almost completely lost in the HES group after 135 min perfusion. In our previous study, enhanced sub-epithelial gaps and enlarged intercellular spaces were also evident in the stroma of most villi after HES perfusion, confirming our current results [19]. However, in our previous work [19], the histological stability score was not significantly different between the Alb and HES perfusion group. The observation of sub-epithelial gaps and enlarged intercellular spaces might be related to HES molecules that overcome the endothelial barrier and reside within the interstitial tissue compartment [28, 29]. As HES is osmotically active and able to bind large amounts of water [30], the fluid shift from the vascular to the interstitial compartment might be increased, leading to the development of a transient interstitial edema. Supplementation of the HES solution with Alb prevented all negative histomorphological effects of HES and resulted in a histological stability score similar to the one obtained after Alb perfusion. Although HES perfusion resulted in morphological damage, we did not detect any differences in the Caspase-3/7 activities between the groups. These findings suggest that HES induced cellular damage is not mediated via Caspase-3/7 associated pathways. However, several authors have shown that apoptosis in intestinal epithelial cells is detectable in the time frame between 1 and 24 h after the apoptosis inducing intervention was applied [31, 32] and we cannot exclude that the time frame of our experiment was too short to detect changes in Caspase-3/7 activity. Intestinal fatty acid binding protein (I-FABP) represents a commonly accepted marker protein for intestinal epithelial cell damage [33, 34]. The amounts of I-FABP in the luminal effluents were analysed by Western blotting and revealed that in the HES perfusion group most I-FABP is released within the first 30 min after the 60 min Alb equilibration phase. This observation suggests that cell damage already occurs shortly after switching the perfusion buffer to 3 % HES, resulting in a release of I-FABP into the intestinal lumen. This is also substantiated by our above mentioned findings, that after 75 min of HES perfusion, the intestinal epithelium is severely damaged and the histoscore significantly reduced. The decline of I-FABP at later time points might be simply due to the fact that once the “pool” of cellular I-FABP is depleted, no new I-FABP protein will be released into the effluent. Moreover, as our model is based on a single perfusion method in which the perfusate is not re-circulated, a rapid “clearance” of I-FABP will occur.

Concerning the metabolic function of the perfused intestine, our results show a statistically significant reduction of lactose digestion and/or uptake of galactose by the intestinal epithelium during HES perfusion. Adding Alb to the HES containing solution abrogated the adverse effects of HES and even increased galactose uptake compared to the control perfusion with Alb alone. The latter, rather unexpected findings, suggests an increased metabolic activity of the intestine when a perfusion with HES and Alb is performed. However, our results are in accordance with data published by others which suggest that Alb in combination with HES can improve endothelial function [5, 11] and enhance tissue perfusion and oxygenation [35].

### Effects of different colloids on endothelial and epithelial barrier function

Employing a previously established isolated perfused model of the mouse intestine [19], we describe a significantly increased fluid shift from the intestinal vasculature into the luminal and lymphatic compartment of the small intestine during HES perfusion, indicative of a disruption of the endothelial and epithelial barrier function. Our quantifications of vascularly applied FITC-dextran in the luminal effluents additionally confirm the above mentioned findings and strongly suggest adverse effects of HES on the epithelial and endothelial barrier. Besides the negative effects of HES on barrier integrity [5, 19, 36], a higher risk for renal replacement therapy and increased mortality after fluid resuscitation with HES has been described [1, 4] and prompted the EMA Pharmacovigilance Risk Assessment Committee to instruct that HES solutions must no longer be used to treat patients with sepsis, burn injuries or critically ill patients (EMA/606303/2013). Interestingly, in our study, supplementing the HES containing solutions with Alb abrogated all adverse effects of HES on vascular, lymphatic and luminal fluid distribution as well as luminal FITC-dextran transfer, therefore improving endothelial and epithelial barrier function. The different colloidal solutions employed in our study all revealed a similar colloid osmotic pressure, so that the favorable effects of Alb cannot be explained by osmotic pressure differences. Concerning the possible molecular mechanisms underlying the protective effects of Alb, recent studies suggested an important role of the endothelial glycocalyx in the preservation of vascular integrity and regulation of endothelial permeability. Albumin contributes to the endothelial surface layer and stabilizes the glycocalyx by binding tightly to its components [37, 38]. As the glycocalyx also protects the endothelial cells from shear stress of blood flow [37] and mediates mechanotransduction [39], the absence of Alb may lead to an alteration and/or degradation of the glycocalyx. This process will probably be facilitated by the presence of bulky HES molecules, leading to increased barrier dysfunction. Other authors propose that Alb facilitates the solubility of the phospholipid sphingosine-1-phosphate (S1P) in aqueous solutions [40] and that the stability of the glycocalyx depends on the presence the S1P and activation of the S1P receptor [40–42]. S1P induced pathways, which involve Erk1/2 and Akt phosphorylation [43], act on the glycocalyx by suppressing matrix metalloproteinase (MMP) activity, thereby protecting cell surface bound syndecan-1 molecules from cleavage by MMP-9 and MMP-13 [44]. Our preliminary results from syndecan-1 ELISA experiments also revealed an increased shedding and release of syndecan-1 from the glycocalyx in the HES perfusion but not in the Alb perfusion group (data not shown). A recent study by Voyvodic et al. showed that syndecan-1 itself seems to be involved in intracellular signaling events. Shear stress induced phosphorylation of Akt was only observed in wild type endothelial cells, while syndecan-1 knockout cells were unable to elicit Akt mediated signaling [45]. Interestingly, our study also provides evidence for the involvement of pro-survival Akt and also Erk1/2 in Alb and HES mediated signaling events. Compared to the HES perfusion, supplementation with Alb resulted in an increased phosphorylation of Akt and Erk1/2 but not Stat5. Whether or not Akt and Erk1/2 are directly involved in the adverse effects of HES and/or the protective functions of Alb needs to be established in further studies.

### Limitations of the study

Based on its ex vivo character, this study has several model related limitations which should be considered. (i) A vascular cell free perfusion was performed in our study. Therefore, the model does not account for possible effects of systemic immune cells and humoral factors on the described HES and Alb mediated mechanisms. (ii) Perfusion with colloid concentrations below 3 % leads to a metabolic and physiological instability of the ex vivo model, as under these conditions colloid osmotic pressure is considerably reduced. Therefore, in our experimental setting vascular perfusion with 1.5 % Alb or 1.5 % HES alone could not be performed and although our in vitro experiments suggest that HES in concentrations of up to 3 % does not induce cell damage/apoptosis in intestinal epithelial cells, we cannot exclude the possibility that the positive effects of HES (1.5 %) plus Alb (1.5 %) on intestinal barrier function and metabolic activity are rather due to the reduction in HES concentration than to protective effects of Alb. (iii) While in vivo, the vascularly applied HES will be diluted by the existent blood, distributed throughout the organism and the final HES concentration in the blood will be reached rather slowly, in our model the final concentration of HES is established immediately without time delay. (iv) In-vivo HES is metabolized by various organs (e.g. liver, kidney) and has a vascular half-life of about 1.4–1.5 h [46]. These characteristics cannot be fully reflected in our single-pass intestinal perfusion model in which the vascular HES concentration stays constant throughout the experiment. However, the time frame of HES application and its vascular concentration correlate well with the clinical situation. (v) Possible effects mediated by the splanchnic nerves are not reflected in the perfused model of the small intestine, as the innervation is disrupted during organ preparation. However, the autonomously working enteric nervous system is still functioning in our model, as can be seen by the regular peristaltic movements of the perfused intestine [19]. To eliminate some of the model related deficiencies, in further studies the isolated perfused mouse small intestinal model could also be combined with a vascular whole blood perfusion. This approach will give deeper insights into the potential interactions of HES molecules with systemic immune cells as well as the humoral immune system and will help to better reflect the clinical scenario of fluid supplementation in our ex vivo model.

## Conclusion

Taken together, employing an isolated perfused model of the mouse intestine, we show that Alb supplementation abrogates the adverse effects of HES on barrier integrity and metabolic function in the intestine and that the underlying mechanism may involve phosphorylation of Erk1/2 and Akt. Our data suggest that adding Alb to HES containing solutions could have the potential to reduce the adverse effects of HES and improve its suitability in the clinic.

## Additional files

10.1186/s12967-016-0810-3 Effects of different colloid containing solutions on epithelial cell damage and apoptosis. Activity of Caspase-3/7 in intestinal tissue (A), CaCo-2 cells (B) and levels of LDH in CaCo-2 cells (C). An increase in apoptosis is detectable between tissue derived before (t0) and at the end of perfusion (t135), but not between the different perfusion groups (A). Apoptosis (B) and cell damage (C) is induced after incubation of CaCo-2 cells with EGTA (+ Control, 0.6 M, 104 h). No changes in apoptosis or cell damage are evident in the different treatment groups. Bars denote the mean ± SD. A: Albumin (N = 6), HES (N = 6), HES/Alb (N = 6). B and C: + Control (N = 3), Albumin (N = 3), HES (N = 3), HES/Alb (N = 3). ** p < 0.01; *** p < 0.001.
10.1186/s12967-016-0810-3 Effects of different colloid containing solutions on Claudin-3 protein expression. Ratio of Claudin-3 and Actin protein expression in intestinal tissue of the Albumin, HES and HES/Alb group after perfusion. No changes in the protein expression levels of Claudin-3 are evident in the different treatment groups. Bars denote the mean ± SD. Albumin (N = 6), HES (N = 6), HES/Alb (N = 6).
10.1186/s12967-016-0810-3 Supplemental results.

## Abbreviations

Alb: albumin
EMA: European Medicines Agency’s
HES: hydroxyethylstarch
I-FABP: intestinal-fatty acid binding protein
NP-40: tergitol-type NP-40
PAGE: polyacrylamide gel electrophoresis
PVDF: polyvinylidenfluorid
RIPA: radioimmunoprecipitation assay buffer
S1P: sphingosine-1-phosphate
SD: standard deviation
Stat5: signal transducer and activator of transcription 5
TBS: tris-buffered saline
W/d: wet-to-dry ratio
