# Supplementary material for: Adverse effects of hydroxyethyl starch (HES 130/0.4) on intestinal barrier integrity and metabolic function are abrogated by supplementation with Albumin
Source: J Transl Med. 2016 Feb 27;14:60. doi: 10.1186/s12967-016-0810-3 (PMC4769564; doi:10.1186/s12967-016-0810-3)
Supplement: Supplementary file 1 — 10.1186/s12967-016-0810-3 Effects of different colloid containing solutions on epithelial cell damage and apoptosis. Activity of Caspase-3/7 in intestinal tissue (A), CaCo-2 cells (B) and levels of LDH in CaCo-2 cells (C). An increase in apoptosis is detectable between tissue derived before (t0) and at the end of perfusion (t135), but not between the different perfusion groups (A). Apoptosis (B) and cell damage (C) is induced after incubation of CaCo-2 cells with EGTA (+ Control, 0.6 M, 104 h). No changes in apoptosis or cell damage are evident in the different treatment groups. Bars denote the mean ± SD. A: Albumin (N = 6), HES (N = 6), HES/Alb (N = 6). B and C: + Control (N = 3), Albumin (N = 3), HES (N = 3), HES/Alb (N = 3). ** p < 0.01; *** p < 0.001. [file 12967_2016_810_MOESM1_ESM.pdf]

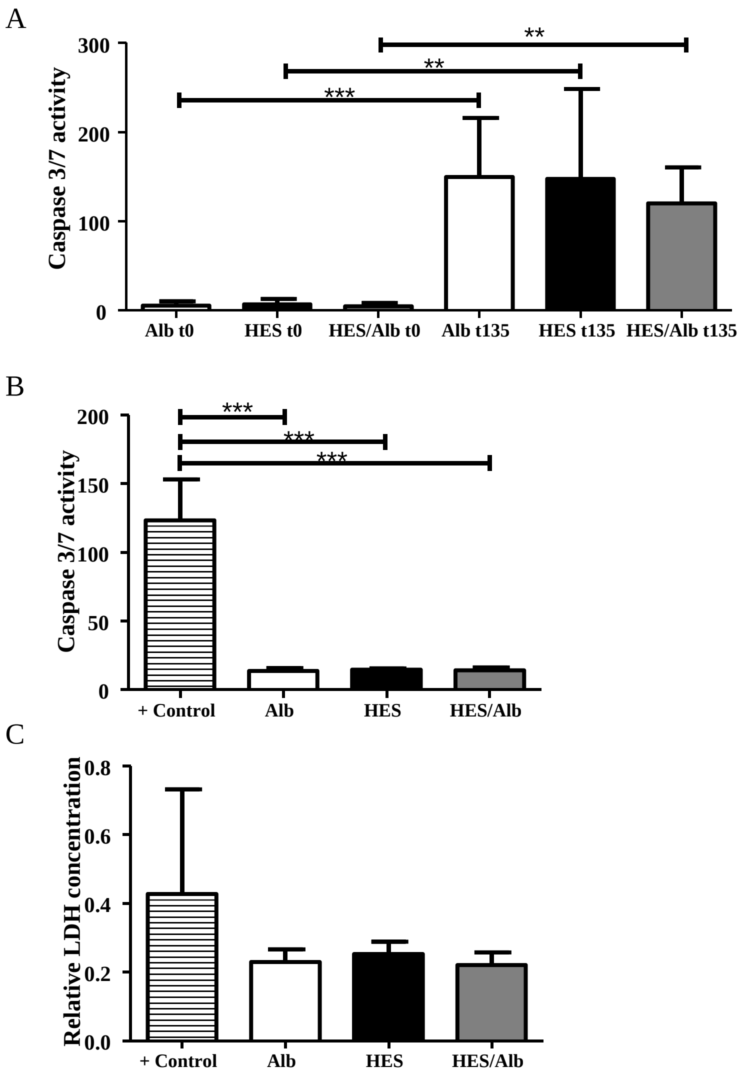

Supplemental Figure 1. Effects of different colloid containing solutions on epithelial cell damage and apoptosis

Activity of Caspase-3/7 in intestinal tissue (A), CaCo-2 cells (B) and levels of LDH in CaCo-2 cells (C). An increase in apoptosis is detectable between tissue derived before (t0) and at the end of perfusion (t135), but not between the different perfusion groups (A). Apoptosis (B) and cell damage (C) is induced after incubation of CaCo-2 cells with EGTA (+ Control, 0.6 M, 104h). No changes in apoptosis or cell damage are evident in the different treatment groups. Bars denote the mean ± SD. A: Albumin (N=6), HES (N=6), HES/Alb (N=6). B and C: + Control (N=3), Albumin (N=3), HES (N=3), HES/Alb (N=3). **, $p<0.01$; ***, $p<0.001$.
