# Supplementary material for: Adverse effects of hydroxyethyl starch (HES 130/0.4) on intestinal barrier integrity and metabolic function are abrogated by supplementation with Albumin
Source: J Transl Med. 2016 Feb 27;14:60. doi: 10.1186/s12967-016-0810-3 (PMC4769564; doi:10.1186/s12967-016-0810-3)
Supplement: Supplementary file 2 — 10.1186/s12967-016-0810-3 Effects of different colloid containing solutions on Claudin-3 protein expression. Ratio of Claudin-3 and Actin protein expression in intestinal tissue of the Albumin, HES and HES/Alb group after perfusion. No changes in the protein expression levels of Claudin-3 are evident in the different treatment groups. Bars denote the mean ± SD. Albumin (N = 6), HES (N = 6), HES/Alb (N = 6). [file 12967_2016_810_MOESM2_ESM.pdf]

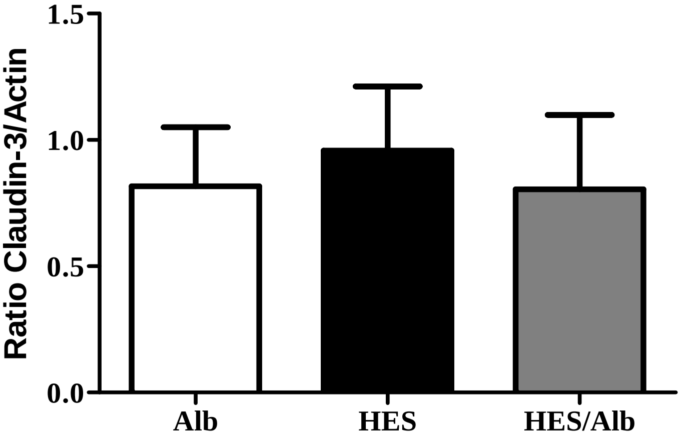

Supplemental Figure 2. Effects of different colloid containing solutions on Claudin-3 protein expression

Ratio of Claudin-3 and Actin protein expression in intestinal tissue of the Albumin, HES and HES/Alb group after perfusion. No changes in the protein expression levels of Claudin-3 are evident in the different treatment groups. Bars denote the mean ± SD. Albumin (N=6), HES (N=6), HES/Alb (N=6).
