# Supplementary material for: Adverse effects of hydroxyethyl starch (HES 130/0.4) on intestinal barrier integrity and metabolic function are abrogated by supplementation with Albumin
Source: J Transl Med. 2016 Feb 27;14:60. doi: 10.1186/s12967-016-0810-3 (PMC4769564; doi:10.1186/s12967-016-0810-3)
Supplement: Supplementary file 3 — 10.1186/s12967-016-0810-3 Supplemental results. [file 12967_2016_810_MOESM3_ESM.pdf]

Supplemental Results:

Histomorphology and cellular damage:

A histological stability score [1] was modified as described in "Methods" and used to evaluate the histomorphological damage of the intestinal epithelium. Perfusion with HES resulted in a significantly reduced histoscore compared to the Albumin (0.29 ± 0.06 vs. 0.53 ± 0.09; p<0.01) or HES/Alb group (0.29 ± 0.06 vs. 0.53 ± 0.08; p<0.01; Fig. 2a). The histomorphological damage in the HES perfusion group was reflected by epithelial shedding and appearance of widened interstitial spaces within the intestinal villi. These changes were already detectable directly at the end of perfusion and could not be observed in the Albumin and HES/Alb group (Fig. 2a). The detection of I-FABP as a marker for intestinal epithelium damage in the luminal effluents emphasizes the negative effects of HES on the epithelial barrier integrity. An increased amount of I-FABP was detected during the first 90 min in the HES perfusion group, however values did not reach statistical significance [HES_{t45-60} (7.34 ± 6.92 a.u.); HES_{t60-75} (14.97 ± 9.27 a.u.); HES_{t75-90} (22.27 ± 12.31 a.u.) vs. Alb_{t45-60} (3.84 ± 6.81 a.u.); Alb_{t60-75} (9.66 ± 11.45 a.u.); Alb_{t75-90} (8.15 ± 9.81 a.u.); p> 0.05; Figure 2B]. Supplementation with Albumin reduced this effect [HES/Alb_{t45-60} (8.03 ± 7.86 a.u.); HES/Alb_{t60-75} (10.66 ± 8.47 a.u.) vs. HES_{t45-60} (7.34 ± 6.92 a.u.); HES_{t60-75} (14.97 ± 9.27 a.u.); p>0.05; Fig. 2b]. For the quantification of apoptosis, a Caspase-3/7 activity assay was employed. The results showed increased apoptosis in intestinal tissue that was obtained at the end of perfusion. These findings are not surprising as the ex-vivo model lacks a functional blood circulation and nerval innervation, which are both important for the survival of the respective tissue and cells. However, no significant differences in apoptosis were observed between the perfusion groups (Additional file 1: Figure 1A). In addition, in-vitro studies employing a commonly accepted model of intestinal epithelial cells (CaCo-2) revealed that even prolonged incubation with high concentrations of HES (3%) did not induce cell damage or apoptosis (Additional file 1: Figure 1B, C), suggesting that the adverse effects of HES on the intestinal epithelium are rather related to an increased pressure (edema formation) caused by HES molecules within the interstitial compartment of the intestine and not due to HES-induced cytotoxicity or apoptosis.

Basic metabolic parameters of the perfused intestine:

Metabolic function of the intestine was evaluated by quantification of the galactose uptake (derived from luminal lactose) and the lactate-to-pyruvate ratio. Both parameters showed a time dependent decrease in all groups, which is related to effects of the ex vivo perfusion procedure on intestinal metabolism (Fig. 3a, b). However, intergroup comparison showed a significant higher reduction of galactose uptake in the HES perfusion group at later time points compared to the Albumin perfusion group [Alb_{t105} (0.54 ± 0.21); Alb_{t120} (0.50 ± 0.13); Alb_{t135} (0.45 ± 0.15) vs. HES_{t105} (0.26 ± 0.08); HES_{t120} (0.13 ± 0.06); HES_{t135} (0.07 ± 0.03);

p<0.05 for t105; p<0.001 for t120 and t135; Fig. 3a]. Interestingly, in the HES/Alb group the luminal galactose uptake was significantly increased compared to the Albumin perfusion, suggesting positive effects of HES/Alb on metabolic functions of the intestine [Alb_{t90} (0.65 ± 0.19); Alb_{t105} (0.54 ± 0.21); Alb_{t135} (0.45 ± 0.15) vs. HES/Alb_{t90} (0.87 ± 0.17); HES/Alb_{t105} (0.80 ± 0.13); HES/Alb_{t135} (0.62 ± 0.08); p<0.05 for t135; p<0.01 for t90 and t105; Fig. 3a].

Regarding the lactate-to-pyruvate ratio, a perfusion related decrease was evident in all groups. There were no significant differences in the time response signatures within or between the groups (p>0.05, intragroup and intergroup comparison; Fig. 3b) and all values were within the physiological range of aerobic metabolism [2]. In addition to the described metabolic parameters, the wet-to-dry ratio (W/d) was calculated. In all groups, W/d was higher at the end of perfusion and compared to a perfusion with Albumin alone, W/d in the HES/Alb group was significantly increased [Alb W/d2 (5.30 ± 0.45) vs. HES/Alb W/d2 (6.09 ± 0.48) p<0.01, Fig. 3c].

Effects of different colloid containing solutions on vascular, lymphatic and luminal flow rates:

For the vascular perfusion with HES the intragroup comparison showed a significantly increased lymphatic flow rate [HES_{t45-60} (1.00 ± 0.00) vs. $HES_{t90-105}$ (3.47 ± 1.13); $HES_{t105-120}$ (4.16 ± 1.42); $HES_{t120-135}$ (4.53 ± 1.77); p<0.01 for t90-105; p<0.001 for t105-120 and t120-135; Fig. 4a], luminal flow rate [HES_{t45-60} (1.00 ± 0.00) vs. $HES_{t90-105}$ (1.77 ± 0.41); $HES_{t105-120}$ (2.25 ± 0.28); $HES_{t120-135}$(2.67 ± 0.24); p<0.001 for all; Fig. 4b] while the vascular flow rate decreased [HES_{t45-60} (1.00 ± 0.00) vs. $HES_{t90-105}$ (0.86 ± 0.06); $HES_{t105-120}$ (0.84 ± 0.07); $HES_{t120-135}$ (0.81 ± 0.06); p<0.001 for all; Fig. 4c].

Vascular, lymphatic and luminal flow rates did not show any statistically significant changes over time in the Albumin and HES/Alb group (intragroup comparison, p>0.05; Fig. 4a-c). Intergroup comparison revealed that HES perfusion significantly increased lymphatic flow [Alb_{t75-90} (1.28 ± 0.21); $Alb_{t90-105}$ (1.47 ± 0.37); $Alb_{t105-120}$ (1.59 ± 0.46); $Alb_{t120-135}$ (1.72 ± 0.57) vs. HES_{t75-90} (2.52 ± 0.66); $HES_{t90-105}$ (3.47 ± 1.13); $HES_{t105-120}$ (4.16 ± 1.42); $HES_{t120-135}$ (4.53 ± 1.77); p< 0.05 for t75-90; p< 0.001 for t90-105, t105-120 and t120-135; Fig. 4a] and luminal flow [Alb_{t75-90} (0.99 ± 0.17); $Alb_{t90-105}$ (1.01 ± 0.11); $Alb_{t105-120}$ (1.06 ± 0.14); $Alb_{t120-135}$ (1.12 ± 0.15) vs. HES_{t75-90} (1.29 ± 0.32); $HES_{t90-105}$ (1.77 ± 0.41); $HES_{t105-120}$ (2.25 ± 0.28); $HES_{t120-135}$ (2.67 ± 0.24); p<0.01 for t75-90; p<0.001 for t90-105, t105-120 and t120-135; Fig. 4b], while vascular flow was reduced [$Alb_{t105-120}$ (0.93 ± 0.03); $Alb_{t120-135}$ (0.93 ± 0.03) vs. $HES_{t105-120}$ (0.84 ± 0.07); $HES_{t120-135}$ (0.81 ± 0.06); p<0.01 for t105-120; p<0.001 for t120-135; Fig. 4c] suggesting that HES perfusion impairs endothelial and epithelial barrier integrity, resulting in fluid shifts towards the lymphatic and luminal compartment. Replacing 1.5 % HES by 1.5 % Albumin abrogated all negative effects of HES on lymphatic flow [HES/Alb_{t75-90} (1.35 ± 0.32); $HES/Alb_{t90-105}$ (1.65 ± 0.67); $HES/Alb_{t105-120}$ (1.79 ± 0.76); $HES/Alb_{t120-135}$ (1.96 ± 0.90) vs. HES_{t75-90} (2.52 ± 0.66); $HES_{t90-105}$ (3.47 ± 1.13); $HES_{t105-120}$

(4.16 ± 1.42); $HES_{t120-135}$ (4.53 ± 1.77); $p<0.05$ for t75-90; $p<0.001$ for t90-105, t105-120 and t120-135; Fig. 4a], luminal flow [HES/Alb_{t75-90} (0.92 ± 0.11); $HES/Alb_{t90-105}$ (0.88 ± 0.14); $HES/Alb_{t105-120}$ (0.91 ± 0.06); $HES/Alb_{t120-135}$ (1.02 ± 0.12) vs. HES_{t75-90} (1.29 ± 0.32); $HES_{t90-105}$ (1.77 ± 0.41); $HES_{t105-120}$ (2.25 ± 0.28); $HES_{t120-135}$ (2.67 ± 0.24); $p<0.01$ for t75-90; $p<0.001$ for t90-105, t105-120 and t120-135; Fig. 4b] and vascular flow [$HES/Alb_{t90-105}$ (0.95 ± 0.09); $HES/Alb_{t105-120}$ (0.99 ± 0.04); $HES/Alb_{t120-135}$ (0.97 ± 0.04) vs. $HES_{t90-105}$ (0.86 ± 0.06); $HES_{t105-120}$ (0.84 ± 0.07); $HES_{t120-135}$ (0.81 ± 0.06); $p<0.01$ for t90-105; $p<0.001$ for t105-120 and t120-135; Fig. 4c]. No statistically significant differences were detected between the Albumin and HES/Alb group concerning the lymphatic, luminal and vascular flow ($p>0.05$; Fig. 4a-c).

Effects of different colloid containing solutions on endothelial and epithelial barrier integrity:

As surrogate parameter for endothelial and epithelial barrier dysfunction, we determined the translocation of vascularly applied FITC-dextran into the luminal compartment. Perfusion with HES significantly and time dependently increased the luminal FITC-dextran concentrations [HES_{t45-60} (2.77 ± 0.82 $\mu g \cdot ml^{-1}$) vs. $HES_{t90-105}$ (11.72 ± 4.52 $\mu g \cdot ml^{-1}$); $HES_{t105-120}$ (16.09 ± 3.99 $\mu g \cdot ml^{-1}$); $HES_{t120-135}$ (20.13 ± 3.57 $\mu g \cdot ml^{-1}$); $p<0.001$ for all; Fig. 5], whereas the FITC-dextran translocation into the luminal compartment remained unchanged in the Albumin and HES/Alb group ($p>0.05$; Fig. 5). Compared to Albumin perfusion a significantly increased luminal FITC-dextran concentration was observed during HES perfusion [Alb_{t75-90} (1.15 ± 1.11 $\mu g \cdot ml^{-1}$); $Alb_{t90-105}$ (1.25 ± 1.19 $\mu g \cdot ml^{-1}$); $Alb_{t105-120}$ (1.54 ± 1.50 $\mu g \cdot ml^{-1}$); $Alb_{t120-135}$ (1.89 ± 1.87 $\mu g \cdot ml^{-1}$) vs. HES_{t75-90} (5.76 ± 3.23 $\mu g \cdot ml^{-1}$); $HES_{t90-105}$ (11.72 ± 4.52 $\mu g \cdot ml^{-1}$); $HES_{t105-120}$ (16.09 ± 3.99 $\mu g \cdot ml^{-1}$); $HES_{t120-135}$ (20.13 ± 3.57 $\mu g \cdot ml^{-1}$); $p<0.01$ for t75-90; $p<0.001$ for t90-105, t105-120 and t120-135; Fig. 5]. Replacing 1.5 % HES by 1.5 % Albumin abrogated the HES associated translocation of vascularly applied FITC-dextran into the luminal compartment [$HES/Alb_{t90-105}$ (3.21 ± 1.14 $\mu g \cdot ml^{-1}$); $HES/Alb_{t105-120}$ (3.42 ± 1.77 $\mu g \cdot ml^{-1}$); $HES/Alb_{t120-135}$ (3.85 ± 2.83 $\mu g \cdot ml^{-1}$) vs. $HES_{t90-105}$ (11.72 ± 4.52 $\mu g \cdot ml^{-1}$); $HES_{t105-120}$ (16.09 ± 3.99 $\mu g \cdot ml^{-1}$); $HES_{t120-135}$ (20.13 ± 3.57 $\mu g \cdot ml^{-1}$); $p<0.001$ for all; Fig. 5]. Concerning the FITC-dextran translocation into the luminal compartment, no statistically significant differences were detected between the Albumin and HES/Alb group ($p>0.05$; Fig. 5).

Evaluation of the expression levels of the tight junctions protein Claudin-3 in the intestinal tissue derived after perfusion did not reveal any differences between the groups ($p>0.05$; Additional file 2: Figure 2).

Effects of different colloid containing solutions on phosphorylation of key signaling molecules:

Compared to the perfusion with Albumin, the phosphorylation patterns of Erk1/2, Akt and Stat5 were altered in the HES/Alb group [Alb (1.00 ± 0.00) vs. $HES/Alb_{pErk1/2/Erk1/2}$ (2.47 ± 0.28); $HES/Alb_{pAkt/Akt}$ (1.60 ± 0.21); $HES/Alb_{pStat5/Stat5}$ (0.91 ± 0.04); $p<0.01$ for pStat5/Stat5; $p<0.01$ for pAkt/Akt; $p<0.001$ for pErk1/2/Erk1/2; Fig. 6]. In the HES group, only the phosphorylation patterns of Akt and Stat5 were changed [Alb (1.00 ± 0.00) vs. $HES_{pAkt/Akt}$ (0.67 ± 0.30); $HES_{pStat5/Stat5}$ (0.85 ± 0.08); $p<0.05$ for pAkt/Akt; $p<0.01$ for pStat5/Stat5; Fig. 6]. Comparing the phosphorylation of Erk1/2, Akt and Stat5 between the HES perfusion and the HES/Alb perfusion groups showed a significant increase of Erk1/2 and Akt but not of Stat5 phosphorylation in the HES/Alb group [$HES/Alb_{pErk1/2/Erk1/2}$ (2.47 ± 0.28); $HES/Alb_{pAkt/Akt}$ (1.60 ± 0.21); $HES/Alb_{pStat5/Stat5}$ (0.91 ± 0.04) vs. $HES_{pErk1/2/Erk1/2}$ (1.00 ± 0.30); $HES_{pAkt/Akt}$ (0.67 ± 0.30); $HES_{pStat5/Stat5}$ (0.85 ± 0.08); $p<0.001$ for pErk1/2/Erk1/2 and pAkt/Akt; $p>0.05$ for pStat5/Stat5; Fig. 6].

1. Lautenschlager I, Dombrowsky H, Frerichs I, Kuchenbecker SC, Bade S, Schultz H, Zabel P, Scholz J, Weiler N, Uhlig S: **A model of the isolated perfused rat small intestine.** *Am J Physiol Gastrointest Liver Physiol* 2010, **298:**G304-313.
2. Burchardi H: *Die Intensivmedizin.* Springer; 2011.
